# Expression of Concern: Role of Microvascular endothelial cells on proliferation, migration and adhesion of hematopoietic stem cells

**DOI:** 10.1042/BSR-20192104_EOC

**Published:** 2020-07-02

**Keywords:** Cell adhesion, Cell migration, Cell proliferation, Chemotaxis, Hematopoietic stem cells, Microvascular endothelial cells

The Editorial Office has been made aware of potential issues surrounding the scientific validity of this paper, hence has issued an expression of concern to notify readers whilst the Editorial Office investigates.

